# Evaluating the Effectiveness of Cultural Education Training: Cultural Competence and Cultural Intelligence Development among Nursing Students

**DOI:** 10.3390/ijerph18084002

**Published:** 2021-04-11

**Authors:** Anna Majda, Joanna Zalewska-Puchała, Iwona Bodys-Cupak, Anna Kurowska, Krystian Barzykowski

**Affiliations:** 1 Faculty of Health Sciences, Medical College, Jagiellonian University,12 Michalowskiego Str., 31-007 Krakow, Poland; anna.majda@uj.edu.pl (A.M.); j.zalewska-puchala@uj.edu.pl (J.Z.-P.); anna2.kurowska@uj.edu.pl (A.K.); 2 Institute of Psychology, Jagiellonian University, 6 Ingardena Str., 30-060 Krakow, Poland; krystian.barzykowski@uj.edu.pl

**Keywords:** cultural competence, cultural intelligence, assessment of cultural competence, assessment of cultural intelligence, nursing students at a master’s level

## Abstract

Background: Since 2012, education standards in medical faculties in Poland have allowed medical universities to introduce content related to multiculturalism. On the one hand, this creates a necessity to introduce new strategies, forms, and techniques of education aimed at the development of knowledge, skills, and attitudes in terms of multiculturalism. On the other hand, there is a need to evaluate their effects. The main goal of this study was to evaluate the cultural competence and cultural intelligence of master’s degree nursing students before the commencement of and two months after cultural education training in the form of the intercultural communication workshops included in the study program. Methods: The following questionnaires were used in the study: the Cross-Cultural Competence Inventory (CCCI) and the Cultural Intelligence Scale (CQS). Two consecutive classes (2019 and 2020) of master’s nursing students were tested twice (pre-test, post-test). The study was conducted at a leading medical university that educates nurses at a master’s level in Poland. In total, 130 master’s nursing students took part in this evaluative study: 64 individuals in 2019 (study 1) and 66 individuals in 2020 (study 2). Results: In comparison to the pre-test, the post-test showed that the surveyed students in both study 1 and study 2 obtained significantly higher overall results in terms of cultural intelligence (*p* = 0.001; *p* = 0.004, respectively) as well as in the behavioral (*p* = 0.001; *p* = 0.002) and cognitive (*p* = 0.001; *p* = 0.008, respectively) subscales. The cultural competence results were also higher overall, but the difference was insignificant. Conclusions: The study shows the efficiency of training/workshops in the development of culturally specific knowledge and cultural intervention skills. At the same time, it postulates the need to plan and organize cultural education programs in a form that aims to improve the development of culturally sensitive attitudes.

## 1. Introduction

The development of intercultural competence can be considered from the perspectives of two categories: cultural competence and cultural intelligence. At present, these are indispensable for everyone, including medical professionals, nurses, and nursing students.

Cultural competence is seen as an essential element when it comes to providing effective and culturally appropriate health services [1,2]. Cultural competence reduces racial and ethnic differences in health and care, and it improves the quality of healthcare, patient satisfaction, and health outcomes [3]. Over the past three decades in nursing, several models of cultural competence have been developed. As previously, extensively reviewed and presented by Loftin et al. [4] and Matsumoto and Hwang [5], work has been undertaken to develop instruments for measuring cultural competence and empirically testing it in terms of psychometric assessment [6,7,8]. The concept of cultural intelligence was introduced to science at the beginning of the 21st century and was initially present mainly in scholarship related to social psychology, business, and management of multicultural international teams [9]. It is rarely used to explain social phenomena in the area of healthcare [10,11,12].

Currently in Poland, due to globalization, there are more foreigners working in international teams and more frequent contact between medical professionals and patients with different cultural backgrounds; therefore, there has been an increased interest in issues of multiculturalism and in the development and measurement of cultural competence and cultural intelligence [13]. Considering the need to use proven and reliable research instruments, procedures have been introduced for the adaptation and validation of instruments that measure cultural competence and cultural intelligence, such as the Cross-Cultural Competence Inventory (CCCI) [14], the Cultural Intelligence Scale (CQS) [15] and the Polish version of the Nurse Cultural Competence Scale (NCCS-P) [16].

In order to effectively interact with different clients in different situations, nurses require appropriate therapeutic and intercultural communication skills [17]. Recommendations have been made around the world to include cultural information in the study programs of nursing students to better prepare them for diverse populations [18]. However, the inclusion of cultural aspects in educational programs has been insufficiently researched [12,19,20,21]. It is difficult to find studies based on the use of CQS and CCCI instruments to measure the cultural intelligence and cultural competencies of nursing students. However, attention is rarely drawn to the three areas of education outcomes (i.e., knowledge, awareness and sensitivity, skills and behavior), which may be subject to changes in intercultural communication training, the effect of which is the development of cultural competence and cultural intelligence. As a result, the aforementioned areas are treated separately.

Since 1999, Poland has implemented changes in the education of nurses as a result of the Bologna Process and the recommendations of European Union directives (Directive 2005/36/EC for the recognition of professional qualifications; modified in 2013—Directive 2013/55/EU). The basic standards of nursing education are contained in the legal document, the Regulation of the Minister of Science and Higher Education of 9 May 2012, describing the education standards for medicine, dentistry, pharmacy, nursing, and obstetrics (Journal of Laws 2012, item 631). This made it possible for medical universities in Poland to introduce content related to multiculturalism in medicine and nursing.

Understanding intercultural communication competence (ICC) leads to offering equal opportunities for those to whom healthcare services are provided [20]. Problematic communication in the relationships between nurses and patients contributes to healthcare inequalities, lack of cultural safety for patients, distrust, unavailability of health care, dissatisfaction with care, and adverse health outcomes [22]. The learning experiences of ICC nursing students should be examined when determining the effectiveness and appropriateness of the inclusion of ICC in nursing study programs [18] and in master’s degrees in Poland. Education in the field of ICC is intended to provide nursing students with the skills, knowledge, and attitudes required to provide appropriate care to culturally diverse clients [23]. However, there is limited evidence on the impact of the introduction of ICC on culture- and communication-oriented training for nursing students. In this study, the cultural competence and cultural intelligence of nursing students in Poland were assessed before and after training in the form of intercultural communication workshops under a master’s degree program. In particular, the study aimed to determine whether multiculturalism workshops differ in terms of their effectiveness in shaping knowledge, skills, and attitudes.

The main goal was to evaluate the cultural competence and cultural intelligence of master’s nursing students before the commencement of and two months after cultural education training in the form of the intercultural communication workshops included in the study program.

## 2. Materials and Methods

### 2.1. Design and Data

The study was conducted at a leading medical university that educates nurses at a bachelor’s and master’s level in Poland. A within-subject design was employed in the present study. The total score of the CQS (with the results in the CQS subscales) and the total score of the CCCI (with the results in the CCCI subscales) were analyzed before and after the workshop.

The study was longitudinal, targeted, cross-sectional, and a sample was convenient. Only nursing students in their first year of a master’s degree were included in the study. They were enrolled in a regular master’s program. All subjects completed nursing studies at the bachelor’s level. The set of research instruments was prepared in printed form. In total, 264 questionnaires were distributed (132 before and 132 after the workshops). They were distributed by individuals who were not involved in teaching the students. Data were collected in January 2019 (study 1) and January 2020 (study 2) before workshops on intercultural communication and two months after their completion in March 2019 (study 1) and March 2020 (study 2).

### 2.2. Sample

Taking into account that not all students participated in the study, the final sample consisted of 130 participants (64 in study 1 and 66 in study 2). The criterion for inclusion in the first measurement, i.e., the pre-test, was the completion of undergraduate studies in the field of nursing and passing the “Theories of Nursing” course. The criteria for inclusion in the second measurement, i.e., the post-test, were the completion of the questionnaires before the workshops and participation in the intercultural communication workshops. The exclusion criteria in the first measurement were the completion of undergraduate studies in a medical field other than nursing and not passing the “Theories of Nursing” course. The exclusion criterion in the second measurement was a failure to participate in the workshops on intercultural communication.

Briefly, 64 nursing students in the first year of their master’s degree studies participated in study 1 in 2019 and 66 students participated in study 2 in 2020. Therefore, there was a total of 130 participants before the training in the form of intercultural communication workshops (4 men—0.03%; M age = 23.57, SD = 1.75) (99% of all first-year students). Both groups were similar in age, gender, level of studies, terms of the lack of participation in cultural training, direct contact with foreigners, and travel outside of Poland for more than one year. Both study 1 and study 2 were conducted twice (the first measurement constituted the pre-test before the start of the workshop/training; the second measurement constituted the post-test two months after the end of the workshop/training) to compare the cultural competence and cultural intelligence of nursing students before and after the workshop/training for two consecutive years. Two months after the end of the intercultural communication workshops/training, a study of the same 130 students took place in 2019 and 2020. First, we describe the results of study 1, followed by the results of study 2.

The required sample sizes for the main analyses were determined by means of G*Power software [24]. The sample size analysis was performed assuming a desired power of 80%. As elaborated below (Section 2.5), dependent *t*-tests will be used to analyze the differences between sessions. The required sample sizes for 80% power for small, medium, and large effect sizes (phi: 0.1, 0.3, 0.5, respectively) are 786, 90, and 34, respectively. Therefore, a sample size of 64 and 66 in study 1 and study 2, respectively, allowed very good power and good power to detect large- and medium-sized effects, respectively, for the main analyses.

### 2.3. Measurement Tool

The following research instruments were used in the present study.

*The Cross-Cultural Competence Inventory (CCCI)* [6], adapted to the Polish language [14]. The CCCI is a broad, multidimensional instrument for the in-depth and comprehensive measurement of cultural competence. The CCCI also touches on deeper layers of competence: attitudes of cultural sensitivity/awareness, cultural skills, and the application and use of cultural knowledge. The CCCI measures three aspects of cultural competence: cognitive, emotional, and behavioral. This is particularly important considering the fact that the most commonly used definition of cultural competence refers directly to three areas: knowledge—providing culturally specific information; skills—involving multicultural interventions; attitudes—cultural empathy, openness, curiosity, tolerance, lack of prejudice in interpersonal relationships, awareness of one’s own value system and its limitations, and an awareness of different perspectives and hierarchies of values, norms and patterns of behavior [25]. The instrument consists of 58 items relating to the following 6 subscales: (1) cultural adaptation—18 items; (2) self-presentation—4 items; (3) ambiguity/uncertainty tolerance—11 items; (4) determination—7 items; (5) readiness to engage—11 items; (6) mission—7 items, and a five-item scale of lies and social approval, treated as a control scale assessing the need to be socially accepted. Answers were provided with the use of a six-point Likert scale, where 6 meant “I strongly agree” and 1 meant “I strongly disagree.” Cultural competence helps achieve effective communication between people of different cultures. The CCCI obtained satisfactory psychometric properties and reliability (internal consistency of Cronbach’s alpha 0.70 to 0.94) [6]. Similarly, satisfactory results were obtained in a Polish study (internal consistency of Cronbach’s alpha 0.83 to 0.86) [14].

*The Cultural Intelligence Scale (CQS)* [9], adapted to the Polish language [15]. The CQS is a slightly different concept. This scale is not as in-depth as the CCCI; it is a little more cognitive, more focused on examining knowledge, including meta-level knowledge, and less focused on attitude (e.g., mindfulness) and behavior. Similar to other types of intelligence, cultural intelligence is understood as the ability to adapt to the surrounding environment and to interpret unknown and ambiguous behavior. It is defined as the ability to function effectively in an environment that is culturally different from one’s own [9]. The CQS consists of 20 items, the scope of which covers the following 4 subscales: (1) metacognitive CQ; (2) cognitive CQ; (3) motivational CQ; and (4) behavioral CQ. Answers were provided with the use of a seven-point Likert scale, where 7 meant “I strongly agree” and 1 meant “I strongly disagree.” The CQS is characterized by good reliability indices in the range of 0.70–0.86 [9] in international studies. Polish studies have shown that the CQS also has satisfactory psychometric properties: it is characterized by high reliability (Cronbach’s alpha 0.94 to 0.95) and sufficient theoretical and criteria validity [15].

### 2.4. Educational Intervention

Training in the form of the intercultural communication workshops included in a master’s nursing studies curriculum has been described in detail in a separate article [26]. The training mainly used a kinesthetic, sensory learning style [27]. The training was based on the experiential learning cycle by D. Kolb [28] and the model of transcultural nursing by J.N. Giger and R.E. Davidhizar [29]. The workshops included 10 h of practical classes in groups of 18–20 people (once a week for two weeks; each session was 5 h long); subsequently, there were 10 h of lectures (once a week for two weeks; each lecture was 5 h long). During the practical classes, active teaching strategies were used (simulation, role-playing, visualization, cases, didactic games, brainstorming). These strategies were based on experiencing, reflecting, discovering, and engaging. The program of the workshops was designed to improve cultural competencies and cultural intelligence of master’s nursing students in areas of knowledge, skills, and attitudes. The program was based on the applicable standards and learning outcomes. It covered content such as the awareness of one’s own identity and culture; the influence of identity and culture on communication; the influence of cultural baggage and stereotypes on the perception of others; symptoms of culture shock and acculturation strategies; post-traumatic stress disorder in refugees; dimensions of culture; interpreting verbal and non-verbal behavior of representatives of various cultural and religious circles; differences in the provision of care for people with different cultural backgrounds; and micro-inequality and micro-affirmative behavior.

### 2.5. Data Analysis

The software STATISTICA (version 12.0; Site License) was used for statistical analysis. We conducted a series of dependent *t*-tests on the total score in the CCCI and CQS (also on total scores within each subscale of the questionnaires) to find differences between the first session (before the training) and the second session (after the training). For all statistical tests (Student’s *t*-test) reported below, the rejection was set to a significance level of 0.05 (unless otherwise specified, see below). For all *t*-tests, the effect size was measured by Cohen’s *d* with small, medium, and large effects defined as 0.2, 0.5, and 0.8, respectively [30]. To control for multiple comparisons, we chose the false discovery rate correction [31], which was applied when the difference reached the level of statistical significance. Two study groups were compared, using an independent sample *t*-test on all subscales of the CCCI and CQS. There were no significant differences between groups on all of the subscales (*p* > 0.078) but one, namely, the mission measured before the workshop. More precisely, participants in study 1 scored lower (M = 30.46, SD = 4.26) than participants in study 2 (M = 31.97, SD = 3.41). The required sample sizes for the main analyses were determined by means of G*Power software [24]. The sample size analysis was performed assuming a desired power of 80%. Dependent *t*-tests were used to analyze the differences between sessions. The required sample sizes for 80% power for small, medium, and large effect sizes (phi: 0.1, 0.3, 0.5, respectively) is 786, 90, and 34, respectively. Therefore, a sample size of 64 and 66 in study 1 and study 2, respectively, allowed very good power and good power to detect large- and medium-sized effects, respectively, for the main analyses.

### 2.6. Ethical Considerations

The study design was positively received by the Bioethics Committee (No. KE/03/012018). The study was developed and conducted in accordance with: (1) the principles of Good Scientific Practice; (2) the Act of 10 May 2018 on the Protection of Personal Data; (3) the principles of the Helsinki Declaration and (4) Regulation (EU) 2016/679 of the European Parliament and of the Council of 27 April 2016, on the protection of individuals with regard to the processing of personal data. The participants of the study were provided with all the necessary information about the study, its purpose, and its procedures. Each participant received oral and written information about the purpose of the study and that participation in the study was voluntary. Importantly, all participants were guaranteed the right to withdraw from participation in the study at any time without giving a reason or suffering any consequences. In order to maintain anonymity, identification marks were provided on the research instruments. Submitting the completed questionnaires was tantamount to consent to participate in the study.

## 3. Results

### 3.1. Study 1

#### 3.1.1. Cultural Intelligence Scale (CQS)

The overall means for the CQS (and each subscale) in session 1 and session 2 are provided in Table 1. The Student’s *t*-test for dependent samples showed that the results of the nursing students in study 1 were significantly higher after participating in the intercultural communication workshops on cultural intelligence than before them. In study 1, the workshops significantly increased the cultural intelligence of the students who were the subject of the study in all subscales (in order): behavioral, cognitive, motivational, and metacognitive. The effect size (Cohen’s *d*) was large; it was medium in the motivational subscale.

#### 3.1.2. Cross-Cultural Competence Inventory (CCCI)

The overall means for the CCCI (and for each subscale) in session 1 and session 2 are provided in Table 2. The Student’s *t*-test for dependent samples showed that the overall results and the results for individual subscales of nursing students in terms of cultural competence in study 1 were higher but statistically insignificant after participating in the intercultural communication workshops. The results indicate that the workshops in study 1 provided nursing students with the opportunity to engage in intercultural communication (*p* = 0.068), although this difference did not reach the level of statistical significance.

### 3.2. Study 2

Study 2 was conducted in 2020 to replicate the findings of study 1 with a different population of students but with the same training program.

#### 3.2.1. Cultural Intelligence Scale (CQS)

The overall means for the CQS (and each subscale) in session 1 and session 2 are provided in Table 3. The Student’s *t*-test for dependent samples showed that, in study 2, the overall results of nursing students in terms of cultural intelligence were significantly higher after participating in the intercultural communication workshops. In study 2, the workshops also increased all the results on the cultural intelligence subscales of the students who participated in the study; however, the impact was only statistically significant in the behavioral and cognitive subscales. While the effect sizes (Cohen’s *d*) were in between small (0.2) and medium (0.5) for most of the results, it was unequivocally small on the motivational subscale.

#### 3.2.2. Cross-Cultural Competence Inventory (CCCI)

The overall means for the CCCI (and for each subscale) in session 1 and session 2 are provided in Table 4. The Student’s *t*-test for dependent samples showed that after the nursing students participated in the intercultural communication workshops, the means for the total CCCI score and CCCI subscales, in general, increased, but not significantly from the pre-test to the post-test in the second session.

## 4. Discussion

A lack of knowledge, cultural skills, and culturally sensitive attitudes among nurses may contribute to the development of difficulties in building therapeutic relationships with patients and may lead to inequalities in the provision of healthcare services [32]. In order to provide healthcare that is adequate to cultural needs, medical students and healthcare professionals must be properly trained. This study presents for the first time a report on the level of cultural competence and cultural intelligence of Polish nursing students and their development after intercultural communication workshops. The Cross-Cultural Competence Inventory (CCCI) and the Cultural Intelligence Scale (CQS), which are standardized research instruments that were adapted to Polish conditions, were used.

What we see in the presented study is a susceptibility to change as a result of intercultural communication workshops. The overall cultural competence results improved after the workshop, but not significantly. The results on individual subscales (cultural adaptation, self-presentation, ambiguity tolerance, determination, readiness to engage, mission) also increased, but not significantly. The effect size for the difference for general cultural intelligence before and after the workshops in the self-assessment of the respondents was high (study 1) or average (study 2), as well as in individual subscales: metacognitive, cognitive, behavioral, motivational. In summary, Polish nursing students scored higher in terms of knowledge, attitudes, and commitment to knowledge acquisition, as well as in terms of their ability to use culturally appropriate behavior when interacting with people with different cultural backgrounds. The nursing students obtained slightly lower results in terms of the development of culturally sensitive attitudes, which somewhat surprised those who conducted the workshops, as they had made every effort to develop these attitudes. This could be explained by the fact that shaping attitudes requires even more time to apply activating teaching strategies based on experience, motivation, and evaluation of training resulting from a longer-term perspective. Nevertheless, the results provide grounds to consider the workshop program as quite successful in developing cultural intelligence and as helpful in recognizing unknown cultural environments. On the other hand, it seems that the training program requires some refinement in terms of developing cultural competence. Among other things, the training program requires more intensive use of activating teaching strategies, the reconfiguration of practical classes into smaller groups in more intimate conditions, and an increase in the number of hours of practical classes. The training program requires that further attention be paid not only to equipping nursing students with cultural knowledge and the ability to adequately behave in intercultural situations but also to striving to change their attitudes. It also requires a more intense change in the awareness and sensitivity of nursing students to cultural differences. This is a difficult and demanding task.

A study of the cultural competence of nursing students at a bachelor’s level in Saudi Arabia with the use of the Inventory for Assessing the Process of Cultural Competence among Healthcare Professionals—Revised (IAPCC-R) showed that the majority of students were culturally aware and dealt with people from different cultures. In terms of the cultural competence subscales, the students from Saudi Arabia showed greater cultural desires compared to cultural knowledge, despite the availability of content related to cultural knowledge while studying [33]. Other studies confirmed that cultural competence was significantly higher in groups that had educational experience in developing it [11,34]. After receiving training on intercultural communication, Canadian nursing students demonstrated increased levels of motivation, confidence, patience, and willingness to interact with culturally diverse clients. Moreover, they found that knowledge of intercultural communication strengthens empathic, patient-centered experiences [21]. In the presented studies, the mean result of the cultural intelligence of Polish nursing students after participating in the workshops, as measured by CQS, was 85.75 in study 1 and 86.02 in study 2. This was similar to the results obtained with the same CQS but carried out among Iranian nurses, i.e., 88.2 [12]. As many authors point out, a high level of cultural intelligence increases the ability to act beyond cultural barriers [35,36] and reduces stress among nurses [37]. Keyvanara et al. [10] draw attention to the need to develop cultural intelligence and effective methods of teaching CQ among students in Iran.

In the presented studies, the results of the training program seem to indicate a promising investment that is worthy of scholarly attention. Due to the increasing cultural diversity of patients and their expectations, health care requires systematic investment in the development of nurses’ cultural competence [38] and cultural intelligence, as is also confirmed by the results of the presented study.

### 4.1. Strengths and Limitations

A strength of this study was the assertion that it is worth studying changes after training with at least two instruments so that the evaluation of the effects and their strength are more reliably evaluated and measured. We recognize that the main limitations of this study were that it was single-site and had a small sample size. Almost all of the participants were female and young adults, and they potentially had limited encounters with culturally diverse clients. Future research could be extended to many places to include the perspectives of male students, nursing teachers, and participant observation as data sources. We recommend repeating the test on a larger sample.

### 4.2. Study Implications

The study supports current knowledge and the need for formal improvement of the cultural competence and cultural intelligence of nursing students and nurses. In order to provide culturally appropriate healthcare, future healthcare professionals need to be properly trained. The inclusion of aspects of culturally conditioned care in the curriculums of master’s degree nursing students should be a reason for hope; however, it is important to keep in mind that forms, strategies, and techniques of teaching still need improving in order to equip nursing students with cultural competence and cultural intelligence.

## 5. Conclusions

The results of this study suggest that training in the form of workshops affects the knowledge and skills of master’s nursing students. However, in order to shape culturally sensitive attitudes, more intense and profound interactions are necessary. Training in the form of intercultural communication workshops is effective when it comes to increasing knowledge about multicultural issues and cultural intervention skills. Training in the form of intercultural communication workshops shows the need to plan in-depth cultural sensitivity workshops to change master’s nursing students’ attitudes. Despite the small size of the surveyed population, education that focuses on strengthening the cultural competence and cultural intelligence of nursing students is promising and can lead to the creation of competent human resources. For patients with different cultural backgrounds, it can provide better health outcomes, better quality care, and cultural safety. We recommend the training program in the field of intercultural communication as a potential program to be deployed in teaching nursing students at the master’s level.

## Figures and Tables

**Table 1:** Overall results of the Cultural Intelligence Scale (CQS) and subscales of nursing students in 2019 (study 1).

Variable	Pre-Test		Post-Test		*t*	*p*-Value *	*q* (Corrected *p*-Value)	Cohen’s d
	M	SD	M	SD				
CQS—overall result	76.77	19.20	85.75	20.14	−5.61	0.001 *	0.010 *	0.69
Metacognitive CQ	16.03	4.51	18.19	4.82	−4.42	0.001 *	0.020 *	0.55
Cognitive CQ	20.30	5.80	23.00	6.06	−4.26	0.001 *	0.030 *	0.53
Motivational CQ	20.47	6.61	21.86	6.19	−2.91	0.001 *	0.050 *	0.36
Behavioral CQ	19.97	6.70	22.70	6.00	−4.21	0.001 *	0.040 *	0.53

Note. M—mean, SD—standard deviation, Student’s *t*-test, *p*—statistical value, *q*—corrected *p*-value. Tests are statistically significant at the corrected *q* = 0.05 level and were marked by the asterisk ‘*’.

**Table 2:** Overall results of the Cross-Cultural Competence Inventory (CCCI) and subscales of nursing students in 2019 (study 1).

Variable	Pre-Test		Post-Test		*t*	*p*-Value
	M	SD	M	SD		
CCCI—overall result	217.27	25.26	220.51	22.73	−1.56	0.127
Cultural adaptation	77.02	11.48	78.73	11.63	−1.79	0.081
Self-presentation	12.71	3.65	13.16	4.29	−0.94	0.352
Ambiguity/uncertainty tolerance	30.98	7.87	31.20	7.47	−0.31	0.758
Determination	23.78	4.81	23.29	4.67	0.94	0.353
Willingness to engage	42.20	6.48	43.42	5.99	−1.87	0.068
Mission	30.58	4.10	30.71	3.55	−0.27	0.785

Note. M—mean, SD—standard deviation, Student’s *t*-test, *p*—statistical value.

**Table 3:** Overall results of CQS and subscales of nursing students in 2020 (study 2).

Variable	Pretest		Post-Test		*t*	*p*-Value *	*q* (Corrected *p*-Value)	Cohen’s d
	M	SD	M	SD				
CQS—result	79.62	18.39	86.02	20.64	−2.99	0.010	0.004 *	0.37
Metacognitive CQ	17.48	4.80	18.41	4.23	−1.85	0.040	0.069	0.23
Cognitive CQ	20.09	4.74	22.17	6.59	−2.74	0.030	0.008 *	0.34
Motivational CQ	21.15	6.43	21.82	6.19	−1.14	0.050	0.258	0.14
Behavioral CQ	20.89	6.78	23.62	6.35	−3.26	0.020	0.002 *	0.47

Note. M—mean; SD—standard deviation; Student’s *t*-test; *p*—statistical value, * *p* ≤ 0.05; *q*—corrected *p*-value. Tests are statistically significant at the corrected *q* = 0.03 level and were marked by the asterisk ‘*’.

**Table 4:** Overall results of CCCI and subscales of nursing students in 2020 (study 2).

Variable	Pretest		Posttest		*t*	*p*-Value
	M	SD	M	SD		
CCCI—overall result	220.63	20.38	221.02	21.53	0.20	0.845
Cultural adaptation	79.13	10.11	79.00	10.63	0.12	0.904
Self-presentation	12.48	4.04	12.75	3.79	−0.75	0.457
Ambiguity/uncertainty tolerance	30.38	7.00	29.13	6.86	1.75	0.087
Determination	22.69	4.88	23.25	4.44	−1.04	0.304
Willingness to engage	44.08	6.40	45.19	6.02	−1.76	0.084
Mission	31.87	3.50	31.69	4.24	0.37	0.712

Note. M—mean; SD—standard deviation; Student’s *t*-test; *p*—statistical value.

## Data Availability

The data is not publicly archived.
